# Protocatechuic Aldehyde Attenuates Cisplatin-Induced Acute Kidney Injury by Suppressing Nox-Mediated Oxidative Stress and Renal Inflammation

**DOI:** 10.3389/fphar.2016.00479

**Published:** 2016-12-06

**Authors:** Li Gao, Wei-Feng Wu, Lei Dong, Gui-Ling Ren, Hai-Di Li, Qin Yang, Xiao-Feng Li, Tao Xu, Zeng Li, Bao-Ming Wu, Tao-Tao Ma, Cheng Huang, Yan Huang, Lei Zhang, Xiongwen Lv, Jun Li, Xiao-Ming Meng

**Affiliations:** ^1^School of Pharmacy, Anhui Medical UniversityHefei, China; ^2^Anhui Institute of Innovative DrugsHefei, China; ^3^Key Laboratory of Anti-inflammatory and Immune Medicine, Ministry of EducationHefei, China; ^4^Department of Pediatrics, Division of Hematology/Oncology, Aflac Cancer and Blood Disorders Center, Children’s Healthcare of Atlanta, Emory University School of MedicineAtlanta, GA, USA

**Keywords:** protocatechuic aldehyde, acute kidney injury, Nox, oxidative stress, inflammation, necroptosis

## Abstract

Cisplatin is a classic chemotherapeutic agent widely used to treat different types of cancers including ovarian, head and neck, testicular and uterine cervical carcinomas. However, cisplatin induces acute kidney injury by directly triggering an excessive inflammatory response, oxidative stress, and programmed cell death of renal tubular epithelial cells, all of which lead to high mortality rates in patients. In this study, we examined the protective effect of protocatechuic aldehyde (PA) *in vitro* in cisplatin-treated tubular epithelial cells and *in vivo* in cisplatin nephropathy. PA is a monomer of Traditional Chinese Medicine isolated from the root of *S. miltiorrhiza* (Lamiaceae). Results show that PA prevented cisplatin-induced decline of renal function and histological damage, which was confirmed by attenuation of KIM1 in both mRNA and protein levels. Moreover, PA reduced renal inflammation by suppressing oxidative stress and programmed cell death in response to cisplatin, which was further evidenced by *in vitro* data. Of note, PA suppressed NAPDH oxidases, including Nox2 and Nox4, in a dosage-dependent manner. Moreover, silencing Nox4, but not Nox2, removed the inhibitory effect of PA on cisplatin-induced renal injury, indicating that Nox4 may play a pivotal role in mediating the protective effect of PA in cisplatin-induced acute kidney injury. Collectively, our data indicate that PA blocks cisplatin-induced acute kidney injury by suppressing Nox-mediated oxidative stress and renal inflammation without compromising anti-tumor activity of cisplatin. These findings suggest that PA and its derivatives may serve as potential protective agents for cancer patients receiving cisplatin treatment.

## Introduction

Cisplatin is widely used in the treatment of various cancers including ovarian, head and neck, testicular and uterine cervical carcinomas (Pabla and Dong, 2008; Sung et al., 2008). Although regarded as one of most effective chemotherapeutic agents by directly interfering with DNA synthesis and inducing apoptosis, adverse effects such as nephrotoxicity put this promising anti-cancer agent in a precarious position. In fact, approximately 30% of patients experience a marked decline in renal function after a single dose injection of cisplatin (Sung et al., 2008). To this point it is important to prevent cisplatin-induced acute kidney injury, which is growing in clinical significance. Although emerging evidence indicates several pathological mechanisms, including excessive inflammatory response, oxidative stress, apoptosis and death of renal tubular epithelial cells, clear therapeutic targets and effective therapies are still lacking (Sancho-Martinez et al., 2015; Yang et al., 2016; Zuk and Bonventre, 2016).

Traditional Chinese Medicine (TCM) may be a promising and novel avenue to effectively treat human diseases with lower toxicity. Indeed, artemisinin is an impressive example of success in the war against malaria. Previous studies indicated that several TCM monomers, such as resveratrol (Kim et al., 2011), Luteolin (Domitrovic et al., 2013), and Emodin (Liu et al., 2016) relieved cisplatin-induced acute kidney injury in animal models. Our recent findings also revealed that 18β-glycyrrhetinic acid alleviated cisplatin-induced apoptosis of renal tubular epithelial cells by targeting HDAC2/BMP-7 axis (Ma et al., 2016). However, the identification of more efficient and low toxic therapeutic agents needs more research. In our pilot study, we tested 10 potential anti-inflammatory TCM monomers including aloin, barbaloin, icariin, protocatechuic acid, protocatechuic aldehyde (PA), puerarin, sodium houttuyfonate, sophoridine, wogonin, and wogonoside, which have not been investigated in the kidney field or in cisplatin-treated tubular epithelial cells (data not shown). Results presented here show that PA, isolated from the root of *S. miltiorrhiza*, is one of the most powerful protective TCM monomers. It significantly suppressed cisplatin-induced injury of tubular epithelial cells and the inflammatory response. This was possibly mediated by inhibition of oxidative stress and programmed cell death. Moreover, the protective role of PA was further evidenced *in vivo* in cisplatin nephropathy, where it prevented decline of renal function and attenuated renal injury. More importantly, results of MMT assay in three tumor cell lines demonstrated that treatment of PA didn’t alter the anti-tumor property of cisplatin. These findings indicate that PA may be a potential therapeutic agent for preventing cisplatin-induced acute kidney injury.

## Results

### PA Ameliorated Cisplatin-Induced Death in HK2 Cells

**FIGURE 1:**
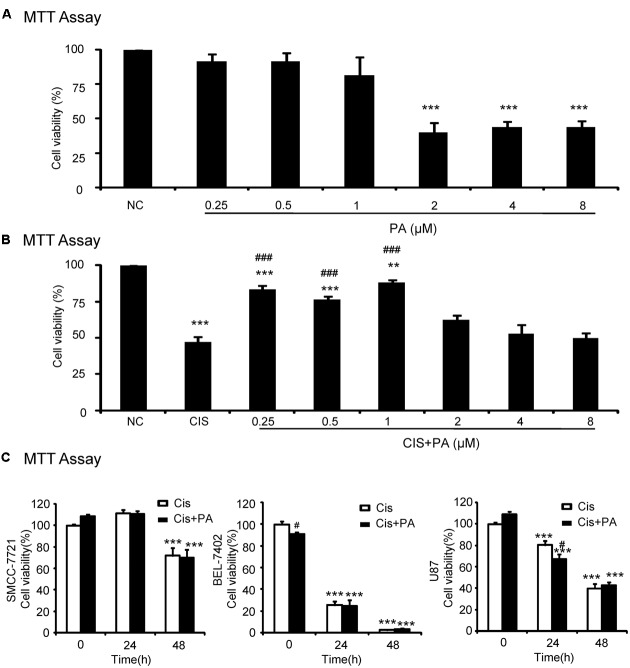
**Effect of protocatechuic aldehyde (PA) on cell viability with or without cisplatin treatment. (A)** Effect of different concentrations of PA on viability of HK2 cells by MTT assay. **(B)** PA restored cell viability in cisplatin-treated HK2 cells (MTT assay). **(C)** Effect of PA on anti-cancer efficacy of cisplatin. Data represent the mean ± SEM for at least 3–4 independent experiments. ^∗^*p* < 0.05, ^∗∗^*p* < 0.01, ^∗∗∗^*p* < 0.001 compared to the control. ^#^*p* < 0.05, ^##^*p* < 0.01, ^###^*p* < 0.001 compared to cisplatin-treated group. Cis, cisplatin; PA, protocatechuic aldehyde.

We used an MTT assay to analyze the impact of PA on cell viability in the human tubular epithelial cell line (HK2). Results show that PA treatment began to reduce cell viability at concentrations greater than 1 μM (**Figure 1A**). Moreover, PA in concentration of 0.25, 0.5, and 1 μM significantly restored cell viability after cisplatin treatment (20 μM; **Figure 1B**). Given these results, we chose 0.25, 0.5, and 1 μM PA for subsequent experiments. We also determined whether PA limited the anti-tumor activity of cisplatin in three solid tumor cells lines, SMCC-7721, BEL-7402, and U87. MTT assay data show that cisplatin reduced cell viability of hepatic cancer SMCC-7721 cells, particularly at 48 h, and administration of PA didn’t reduce the tumor-suppressive effect of cisplatin (**Figure 1C**). This was further supported by the findings that PA didn’t protect against cisplatin-induced tumor cell death in human hepatic cancer line BEL-7402 and malignant gliomaU87 cell line.

### PA Protected against Cisplatin-Induced Cell Damage and Inflammatory Response

**FIGURE 2:**
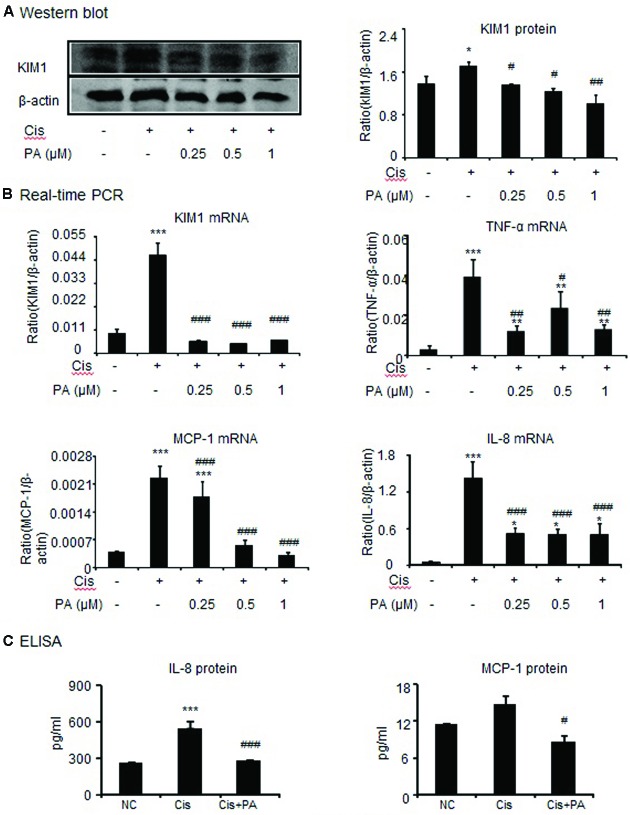
**Protocatechuic aldehyde reduced cisplatin-induced kidney injury molecule-1 (KIM1) level and inflammatory response in HK2 cells. (A)** Western blot analysis and quantitative data of KIM-1 in HK2 cells. **(B)** Real-time PCR in HK2 cells. Results demonstrate that treatment of PA largely reduced cisplatin-induced mRNA levels of KIM1, TNF-α, MCP-1, and IL-8. **(C)** ELISA in HK2 cells. Results indicate that treatment with PA significantly reduced cisplatin-upregulated protein levels of IL-8 and MCP-1 in HK2 cells. Data represent the mean ± SEM for 3–4 independent experiments.^∗^*p* < 0.05, ^∗∗^*p* < 0.01, ^∗∗∗^*p* < 0.001 compared to the control. ^#^*p* < 0.05, ^##^*p* < 0.01, ^###^*p* < 0.001 compared to cisplatin-treated group. Cis, cisplatin; PA, protocatechuic aldehyde.

To assess whether PA reduces kidney damage, we examined mRNA and protein expression of kidney injury molecule-1(KIM1). Western blot and real-time PCR results show that cisplatin upregulated KIM1. This was decreased by PA treatment in a time-dependent manner in HK-2 cells (**Figures 2A,B**). Additionally, real-time PCR and ELISA analysis show that PA protected against inflammatory response as evidenced by decreased chemokine monocyte chemotactic protein (MCP-1), inflammatory cytokine (IL-8), and proinflammatory cytokine TNF-α expression levels (**Figures 2B,C**).

### PA Inhibited Cisplatin-Induced Cell Necroptosis and Apoptosis

**FIGURE 3:**
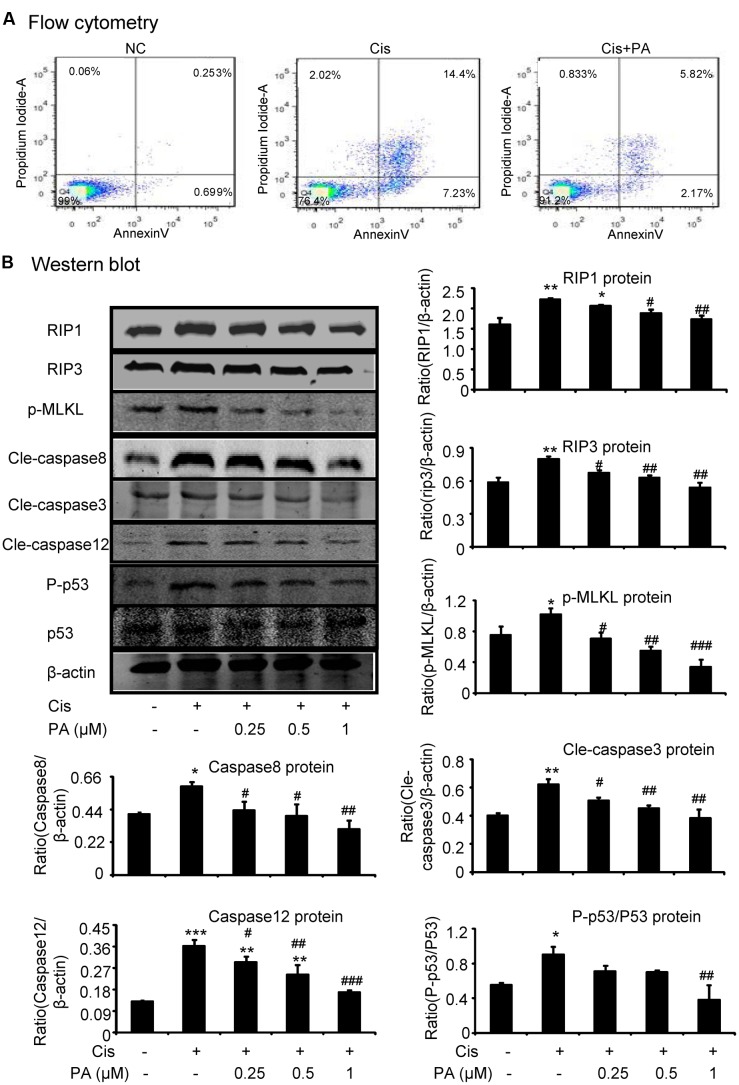
**Protocatechuic aldehyde inhibited cisplatin-induced cell necroptosis and apoptosis in HK2 cells. (A)** Flow cytometry of PI/AnnexinV. Results of flow cytometry demonstrate that PA inhibited cisplatin-induced cell necrosis and apoptosis in HK2 cells; **(B)** Western blot analysis and quantitative data of RIP1, RIP3, p-MLKL in cisplatin-treated HK2. Results show that administration of PA substantially blocked activation of RIP1/RIP3/MLKL axis in cisplatin-stimulated HK2 cells; In addition, results of Western blot and quantitative data demonstrate that treatment of PA gently decreased cisplatin-induced cleavage of caspases and phosphorylation of p53 in HK2 cells; Data represent the mean ± SEM for 3–4 independent experiments. ^∗^*p* < 0.05, ^∗∗^*p* < 0.01, ^∗∗∗^*p* < 0.001 compared to the control. ^#^*p* < 0.05, ^##^*p* < 0.01, ^###^*p* < 0.001 compared to cisplatin-treated group. Cis, cisplatin; PA, protocatechuic aldehyde.

We tested the protective effects of PA on cell death of HK2 by flow cytometric analysis of PI/AnnexinV staining. Results show that PA alleviated cisplatin-induced necroptosis and apoptosis (**Figure 3A**). Mechanistically, PA significantly reduced the key signaling molecules mediating necroptosis, including RIP1, RIP3, and phosphorylation of downstream MLML in HK2 cells (**Figure 3B**). Furthermore, cleaved-caspase-8, cleaved-caspase-3, cleaved-caspase-12, and phosphorylation of p53 were also markedly decreased in response to PA treatment (**Figure 3B**).

### PA Suppressed Cisplatin-Induced Injury *In vitro* via Blocking Nox-Mediated Oxidative Stress

**FIGURE 4:**
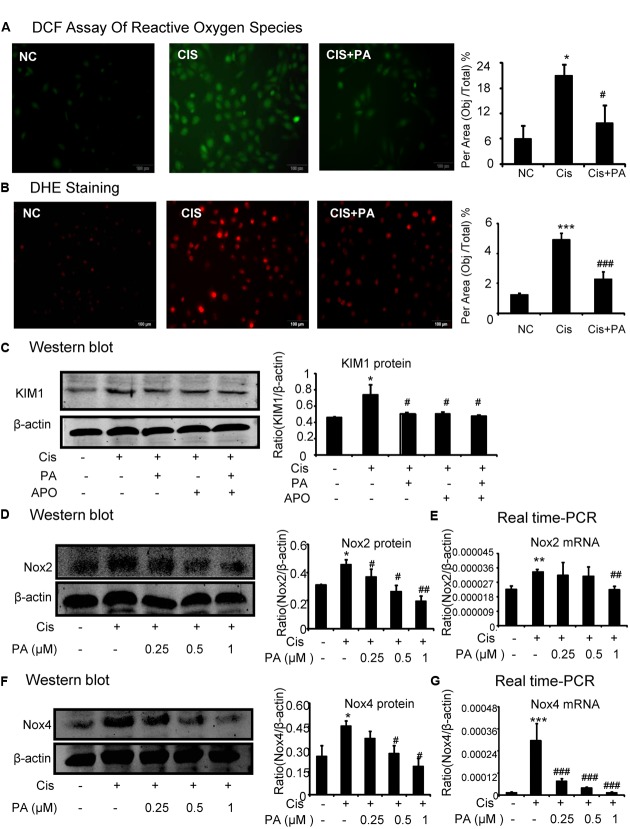
**Protocatechuic aldehyde inhibited cisplatin-induced cell oxidative stress in HK2 cells by suppressing Nox signaling. (A)** DCF Assay of Reactive Oxygen Species. Results of ROS assay demonstrate that PA inhibited cisplatin-induced cell oxidative stress in HK2 cells; **(B)** DHE staining of intracellular ROS levels. PA attenuates cisplatin-induced ROS generation. **(C)** Role of Nox enzymes in the effect of PA on cisplatin-treated HK2 cells. **(D,F)** Western blot analysis and quantitative data of Nox2 and Nox4 in cisplatin-treated HK2 cells. Results show that administration of PA substantially suppressed protein levels of both Nox2 and Nox4 in cisplatin-stimulated HK2 cells. **(E,G)** Real-time PCR in HK2 cells. Results demonstrate that treatment of PA largely reduced cisplatin-induced mRNA levels of Nox2 and Nox4; Data represent the mean ± SEM for 3–4 independent experiments. ^∗^*p* < 0.05, ^∗∗^*p* < 0.01, ^∗∗∗^*p* < 0.001 compared to the control. ^#^*p* < 0.05, ^##^*p* < 0.01, ^###^*p* < 0.001 compared to cisplatin-treated group. Cis, cisplatin; PA, protocatechuic aldehyde; APO, Apocynin.

A reactive oxygen species (ROS) assay was performed using DCF fluorescence in HK2 cells to measure the effect of PA on oxidative stress. Results show that PA largely decreased ROS in cisplatin-stimulated HK2 cells (**Figure 4A**). Moreover, results of DHE staining show that superoxide levels, the reaction product of Nox enzymes, were suppressed by PA treatment (**Figure 4B**). We know that apocynin or PA treatment decreases cisplatin-induced damage of tubular epithelial cells. Interestingly, we found that after blocking Nox enzymes with apocynin, PA failed to further reduce the renal damage (**Figure 4C**). This indicates a role for the Nox family in mediating the protective effect of PA. Therefore, we analyzed the main members of the Nox family in kidney, including Nox2 and Nox4. Western blot results show that PA treatment reduced cisplatin-induced Nox2 and Nox4 protein expression in a dose-dependent manner (**Figures 4D,F**). This was further supported by real-time PCR data showing reduced Nox2 mRNA and Nox4 mRNA (**Figures 4E,G**).

### PA Attenuated Cisplatin-Induced Injury through Nox4-Dependent Mechanisms

**FIGURE 5:**
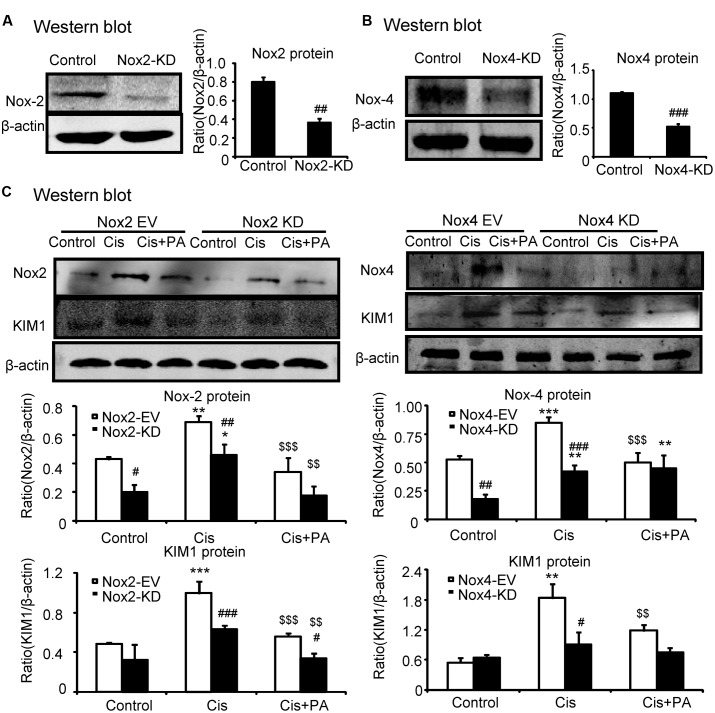
**Protocatechuic aldehyde failed to further reduce cisplatin-induced cell injury in Nox4 knockdown, instead of Nox2 knockdown in HK2 cells. (A,B)** Identification of Nox2 and Nox4 knockdown in HK2 cells; Results show that Nox2 and Nox4 were downregulated by transfection of Nox2 siRNA and Nox4 ShRNA plasmid, respectively. **(C)** Western blot analysis and quantitative data of KIM1 in Nox2 and Nox4 knockdown HK2 cells treated with PA. Data represent the mean ± SEM for 3–4 independent experiments. ^∗^*p* < 0.05, ^∗∗^*p* < 0.01, ^∗∗∗^*p* < 0.001 compared to the control. ^#^*p* < 0.05, ^##^*p* < 0.01, ^###^*p* < 0.001 compared to Nox4 EV group. ^$$^*p* < 0.01, ^$$$^*p* < 0.001 compared to cisplatin-treated group. Cis, cisplatin; PA, protocatechuic aldehyde; EV, empty vector; KD, knockdown.

We determined whether Nox2 and Nox4 are important targets for PA in mediating its protective effects. We found that Nox2 and Nox4 were largely suppressed when shRNA plasmids were transfected into cells (**Figures 5A,B**). Western blot results show that PA further suppressed KIM1 levels in absence of Nox2. But, PA failed to further attenuate renal tubular cell damage when Nox4 was disrupted (**Figure 5C**). This indicates an essential role for Nox4 in mediating protective effects of PA.

### PA Attenuated Cisplatin-Induced Necrosis, Inflammation, and ROS through Nox4-Dependent Mechanisms

**FIGURE 6:**
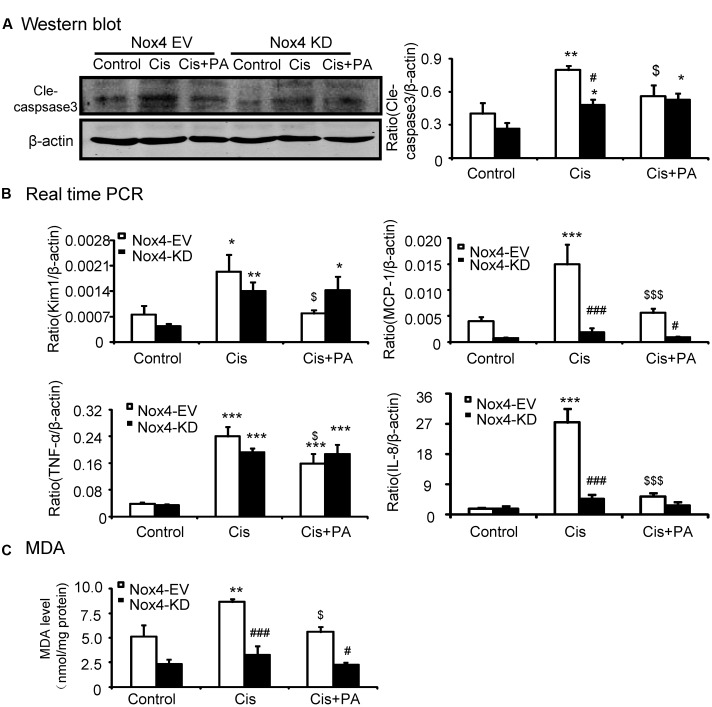
**Protocatechuic aldehyde failed to further reduce cisplatin-induced cell apoptosis, inflammatory response, and oxidative stress in Nox4 disrupted HK2 cells. (A)** Western blot analysis and quantitative data of cleaved-caspase-3. **(B)** Real-time PCR analysis of inflammatory response in Nox4 disrupted HK2 cells. Results show that when Nox4 was disrupted, PA failed to further decrease mRNA levels of KIM1, MCP-1, TNF-α, and IL-8. **(C)** Malondialdehyde (MDA) levels in HK2 cells. Results indicate that when Nox4 was knocked down, PA failed to further decrease the MDA levels in cisplatin-stimulated HK2 cells. Data represent the mean ± SEM for 3–4 independent experiments. ^∗^*p* < 0.05, ^∗∗^*p* < 0.01, ^∗∗∗^*p* < 0.001 compared to the control. ^#^*p* < 0.05, ^##^*p* < 0.01, ^###^*p* < 0.001 compared to Nox4 EV group. ^$^*p* < 0.05, ^$$^*p* < 0.01, ^$$$^*p* < 0.001 compared to cisplatin-treated group. Cis, cisplatin; PA, protocatechuic aldehyde; EV, empty vector; KD, knockdown.

We analyzed the role of PA on the Nox4 pathway in more detail *in vitro*. We found that PA failed to reduce cleaved-caspase-3 levels in Nox4 knockdown HK2 cells (**Figure 6A**). Consistently, when Nox4 was silenced, PA didn’t further suppress the production of inflammatory cytokines (**Figure 6B**). This indicates that PA protects against cisplatin-treated tubular epithelial cells in a Nox-dependent manner. These results were further confirmed by detecting MDA level, which showed that ROS levels were consistent (**Figure 6C**).

### PA Inhibited Cisplatin-Induced Acute Kidney Injury in Mice

**FIGURE 7:**
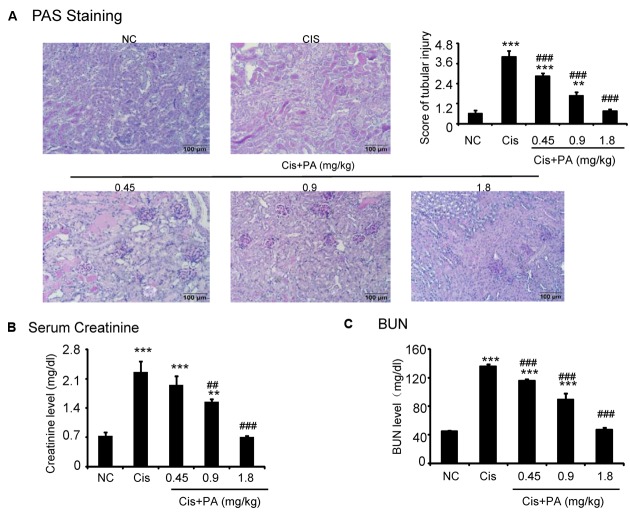
**Protocatechuic aldehyde prevented cisplatin-induced renal injury and decline of renal function *in vivo*. (A)** PAS staining and score. Results of PAS staining and score of severity indicate that treatment of a set concentrations of PA alleviated tubular necrosis, tubular dilation, and cast formation in cisplatin nephropathy; **(B)** Serum Creatinine; **(C)** BUN. Results of serum creatinine and BUN show that treatment of PA restored renal function in cisplatin nephropathy. Data represent the mean ± SEM for 6–8 mice. ^∗∗^*p* < 0.01, ^∗∗∗^*p* < 0.001 compared to control. ^##^*p* < 0.01, ^###^*p* < 0.001 compared to model. Cis, cisplatin; PA, protocatechuic aldehyde.

The protective effect of PA was also evaluated *in vivo* in cisplatin nephropathy. Results of periodic acid-Schiff (PAS) staining revealed that administration of PA in concentrations of 0.45, 0.9, and 1.8 mg/kg reduced tubular necrosis, dilation, and cast formation compared with model groups (**Figure 7A**). The therapeutic effects of PA were further confirmed by detection of renal function, including serum creatinine and blood urea nitrogen. Results show that PA largely prevented the decline of renal function in a dose-dependent manner (**Figures 7B,C**).

### PA Significantly Reduced Cisplatin-Induced Tubular Injury and Inflammation Response in Mice

**FIGURE 8:**
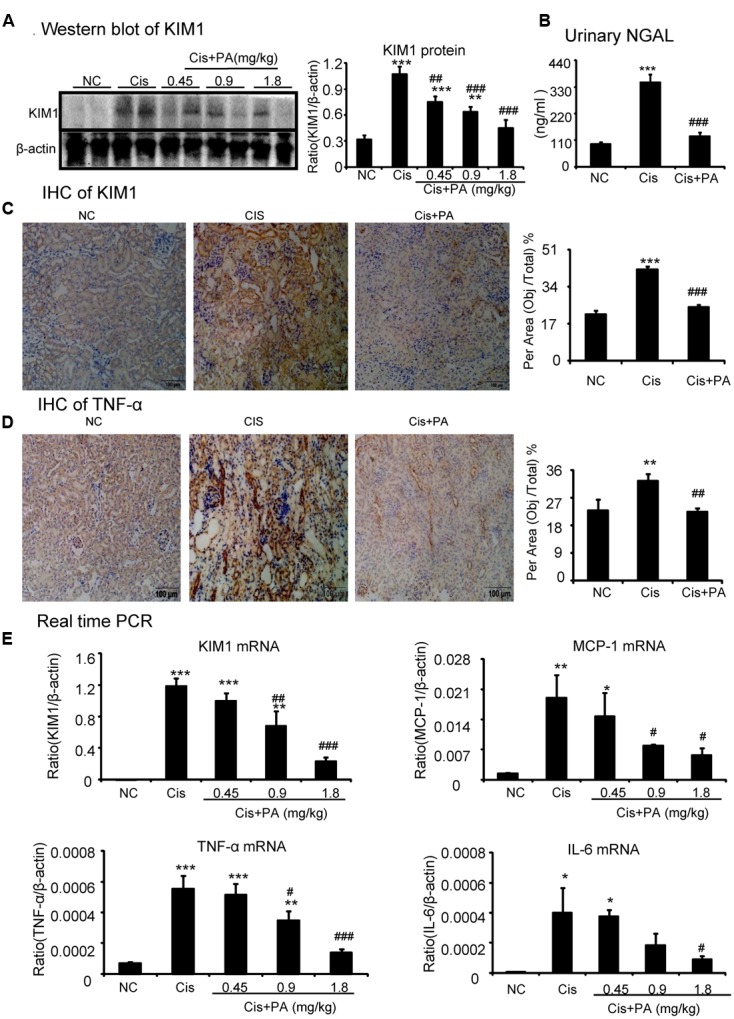
**Protocatechuic aldehyde attenuated cisplatin-induced renal injury and inflammation *in vivo*. (A)** Western blot analysis and quantitative data of KIM1. Results indicate that treatment of PA in a set of concentrations substantially inhibited cisplatin-induced KIM1 in protein levels. **(B)** ELISA of urinary neutrophil gelatinase-associated lipocalin (NGAL). PA treatment also suppressed the increased level of urinary NGAL in cisplatin nephropathy. **(C)** Immunohistochemistry of KIM1. IHC result and quantitative data indicate that treatment of PA reduced the percentage of KIM1+ cells in injured kidney; **(D)** Immunohistochemistry of TNF-α. IHC result and quantitative data indicate that treatment of PA reduced the percentage of TNF-α+ cells in injured kidney; **(E)** Real-time PCR of inflammation indexes. Real-time PCR demonstrate that treatment of PA largely blocked upregulated mRNA levels of KIM1, MCP-1, TNF-α, and IL-6 in cisplatin-injured kidney. Data represent the mean ± SEM for 6–8 mice. ^∗^*p* < 0.05, ^∗∗^*p* < 0.01, ^∗∗∗^*p* < 0.001 compared to control. ^#^*p* < 0.05, ^##^*p* < 0.01, ^###^*p* < 0.001 compared to model. Cis, cisplatin; PA, protocatechuic aldehyde.

Western blot and quantitative data show that KIM1, a key marker for tubular injury, was significantly elevated by cisplatin but reduced by PA treatment in a dose-dependent manner (**Figure 8A**). This was further evidenced by results from IHC and real-time PCR (**Figures 8C,E**). PA treatment also suppressed the increased level of Neutrophil gelatinase-associated lipocalin (NGAL), another kidney injury marker, in urine in cisplatin nephropathy (**Figure 8B**). Additionally, results from IHC show that PA reduced TNF-α positive signals in injured kidney. This was consistent with real-time PCR results showing reduced mRNA levels of proinflammatory cytokines and chemokines including TNF-α, IL-6, and MCP-1 (**Figures 8D,E**).

### PA Protected against Cisplatin Nephropathy by Attenuating Oxidative Stress

**FIGURE 9:**
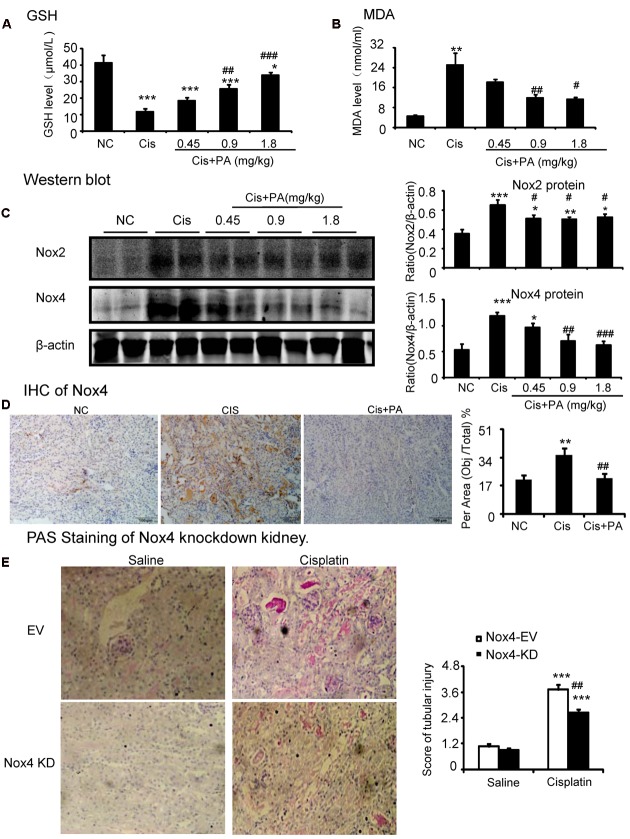
**Protocatechuic aldehyde prevented Nox4-mediated oxidative stress in cisplatin nephropathy. (A)** The changes of MDA levels *in vivo*. **(B)** The changes of glutathione (GSH) levels *in vivo*. Results of the MDA activities and GSH activities show that treatment of PA suppressed renal oxidative stress in cisplatin nephropathy. **(C)** Western blot analysis and quantitative data of Nox2 and Nox4 in cisplatin nephropathy. Results show that treatment of PA in a set of concentrations significantly reduced the protein level of Nox2 and Nox4. **(D)** Immunohistochemistry of Nox4. IHC result and quantitative data indicate that treatment of PA reduced the percentage of Nox4+ cells in injured kidney. **(E)** PAS staining of Nox4 knockdown kidney. PAS staining and score show that lentivirus-mediated knockdown of Nox4 prevented cisplatin-induced kidney damage. Data represent the mean ± SEM for 6–8 mice. ^∗^*p* < 0.05, ^∗∗^*p* < 0.01, ^∗∗∗^*p* < 0.001 compared to control. ^#^*p* < 0.05, ^##^*p* < 0.01, ^###^*p* < 0.001 compared to model. Cis, cisplatin; PA, protocatechuic aldehyde.

We then investigated the underlying mechanisms by which PA improves renal function and limits kidney injury. We found that MDA levels were significantly increased in kidneys of cisplatin-injected mice. But, PA reduced the upregulation of MDA levels in a dose-dependent manner (**Figures 9A,B**). This is consistent with the finding that PA restored the cisplatin-suppressed level of GSH, an antioxidant index. This indicates that PA substantially decreased cisplatin-induced production of ROS. Moreover, Western blot and quantitative data show that PA reduced protein levels of Nox2 and Nox4 in a dosage-dependent manner in cisplatin nephropathy. This was further confirmed by immunohistochemistry that revealed that Nox4 was downregulated in cisplatin-injured kidney treated with PA (**Figures 9C,D**). Additionally, the function of Nox4 was detected *in vivo*. Results of PAS staining indicate that lentivirus-mediated knockdown of Nox4 significantly attenuated cisplatin-induced kidney damage (**Figure 9E**). This demonstrates it may be a critical target to mediate protective effects of PA.

### PA Protected against Cisplatin Nephropathy by Attenuating Cell Death

**FIGURE 10:**
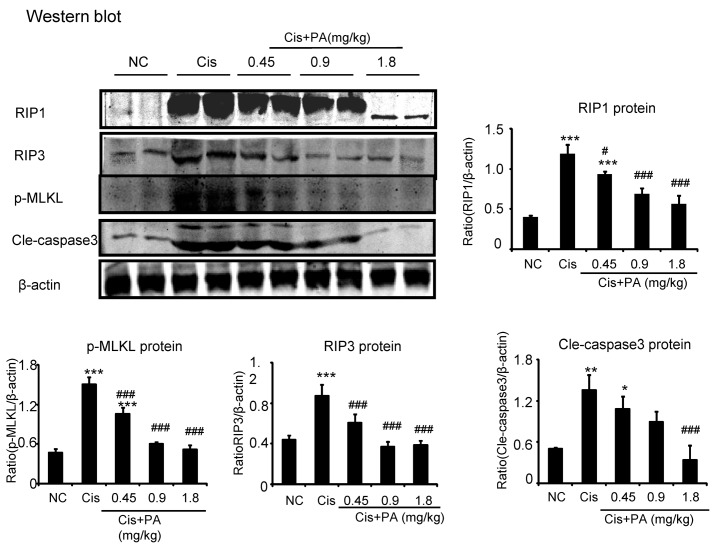
**Protocatechuic aldehyde prevented necrosis and apoptosis in cisplatin nephropathy.** Western blot analysis and quantitative data of RIP1, RIP3, p-MLKL, and cleaved-caspase-3. Data represent the mean ± SEM for 6–8 mice. ^∗^*p* < 0.05, ^∗∗^*p* < 0.01, ^∗∗∗^*p* < 0.001 compared to control. ^#^*p* < 0.05, ^##^*p* < 0.01, ^###^*p* < 0.001 compared to model. Cis, cisplatin; PA, protocatechuic aldehyde.

Our results show that PA suppressed activation of the RIP1/RIP3/MLKL axis, which is regarded as the key pathway mediating necroptosis. We found PA also decreased levels of cleaved-caspase-3 in cisplatin nephropathy (**Figure 10**).

## Materials and Methods

### Murine Model of Cisplatin-Induced AKI

Mice were obtained from Laboratory Animal Center of Anhui province. All animal procedures were approved by the Institutional Animal Experimentation Ethics Committee of Anhui Medical University. Cisplatin with a single dose at 20 mg/kg was injected intraperitoneally in 8-week-old male mice and their littermates injected with saline were set as normal control. Protocatechuic aldehyde (Dalian Meilun Biotech Co. Ltd.), concentrations of 0.45, 0.9, and 1.8 mg/kg, were given via intraperitoneal injection 6 h before cisplatin treatment and injected daily. Mice were sacrificed under anesthesia (10% chloralic hydras by intraperitoneal injection) 3 days after cisplatin injection. Samples of kidney tissues and blood were harvested for further analysis, including BUN (Nanjing Jiancheng Bioengineering Institute) and creatinine, paraffin embedding (Nanjing Jiancheng Bioengineering Institute) and molecular analysis. Paraffin sections (3–5 mm) were stained with Periodic acid Schiff (PAS) Staining kit (Fuzhou Maixin Biotech. Co., Ltd.) and analyzed via immunohistochemistry.

### Reagents and Materials

Antibodies RIP1, RIP3, KIM-1, Nox2, Nox4, TNF-α, and β-actin were purchased from Santa Cruz Biotechnology (Santa Cruz, CA, USA); Rabbit anti-P-MLKL and Anti-cleaved-caspase-3, cleaved-caspase-12 were obtained from Cell Signaling Technology (CST, Danvers, MA, USA). Lipofectamine 3000 was purchased from Science Biotechnology (Invitrogen, Beijing, China). Protein Assay Kit was purchased from Beyotime Institute of Biotechnology (Jiangsu, China). Cell Malondialdehyde (MDA) assay kit (Colorimetric method), reduced glutathione (GSH) assay kit, creatinine (Cr) Assay kit (sarcosine oxidase) and urea (BUN) assay kit were obtained from Nanjing Jiancheng Bioengineering Institute(Nanjing, China). Reactive Oxygen Species Assay (DCF Assay) Kit and Dihydroethidium (DHE) were purchased from Beyotime Institute of Biotechnology (Jiangsu, China). ITC AnnexinV/propidiuiodide was purchased from Bestbio (Shanghai, China). Mouse NGAL ELISA kit was purchased from CUSABIO (Wuhan, China).

### Cell Culture

Solid tumor cell lines (SMCC-7721, BEL-7402, and U87), kidney tubular epithelial cells of human (HK2), and Nox2, Nox4 knockdown HK2 cells were cultured in 5% FBS-containing HyClone^TM^ DMEM/F12 medium at 37°C in humidified 5% CO_2_. After overnight starving in DMEM/F12 medium containing 0.5% FBS, HK2 cells were pretreated with PA (Meilun Biology Technology, Dalian, China) for 6 h before being exposed to cisplatin (20 μM). Twenty-four hour later, the cells were harvested for cell viability, the indexes of kidney injury, oxidative stress, programmed cell death, and inflammatory response such as KIM1, Nox2, Nox4, cleaved-caspase-3, 8, and 12, p-p53, p53, RIP1, RIP3, phospho-MLKL, TNF-α, and MCP-1 using Western blot analysis, real-time PCR or other methods. Three to four *in vitro* experiments were performed independently.

### Cell Viability Assay

Cell viability is determined by MTT assay, according to a purple formazan product produced by mitochondrial dehydrogenase of viable cells. Human HK2 cells were grown in 96-well plates with treatments by a set of concentrations of PA (arranged from 0.25 to 8 μM). After 12 h, the cells were exposed to cisplatin (20 μM) for 24 h in incubator. They were harvested after addition of 5 mg/ml MTT solution to each well for 4 h. Optical density (OD) was determined in microplate reader (Multiskan MK3, Thermo, USA) at 492 nm wavelength.

### RNA Extraction and Real-Time PCR

Total RNA was isolated using the RNeasy Isolation Kit (Qiagen, Valencia, CA, USA) according to the manufacturer’s instructions (Li et al., 2012). The RNA concentration was detected by a NanoDrop 2000 Spectrophotometer (Thermo Scientific, USA). RNA, nuclease-free water and RealMasterMix (Bio-Rad, Hercules, CA, USA) were used for cDNA synthesis. Real-time PCR was performed in a total volume of 9 μl, including 2 μl cDNA solution, 4 μlBio-Rad iQ SYBR Green supermix with Opticon 2 (Bio-Rad, Hercules, CA, USA), 2.4 μl nuclease-free water, and 0.6 μl each primer. The sequences of primers are as follows:

Human IL-8, forward 5′-AGGACAAGAGCCAGGAAGAA-3′,
reverse 5′- ACTGCACCTTCACACAGAGC-3′;
Human TNF-α, forward 5′-CCCAGGGACCTCTCTCTAATCA-3′,
reverse 5′- GCTACAGGCTTGTCACTCGG-3′;
Human KIM-1, forward 5′-CTGCAGGGAGCAATAAGGAG-3′,
reverse 5′-TCCAAAGGCCATCTGAAGAC-3′;
Human Nox4, forward 5′-GGATCACAGAAGGTCCCTAGCAG-3′,
reverse 5′-GCGGCTACATGCACACCTGAGAA-3′;
Human Nox2, forward 5′–3′TTCCAGTGCGTGTTGCTCGAC,
reverse 5′–3′GATGGCGGTGTGCAGTGCTAT;
Human β-actin, forward 5′-CGCCGCCAGCTCACCATG-3′,
reverse 5′-CACGATGGAGGGGAAGACGG-3′;
Mouse IL-6, forward 5′-GAGGATACCACTCCCAACAGACC-3′,
reverse 5′-AAGTGCATCATCGTTGTTCATACA-3′;
Mouse TNF-α, forward 5′- CATCTTCTCAAAATTCGAGTGACAA-3′,
reverse 5′-TGGGAGTAGACAAGGTACAACCC-3′;
Mouse MCP-1, forward 5′- CTTCTGGGCCTGCTGTTCA-3′,
reverse 5′-CCAGCCTACTCATTGGGATCA-3′;
Mouse KIM-1, forward 5′-CAGGGAAGCCGCAGAAAA-3′,
reverse 5′-GAGACACGGAAGGCAACCAC-3′;
Mouse β-actin, forward 5′- CATTGCTGACAGGATGCAGAA-3′,
reverse 5′-ATGGTGCTAGGAGCCAGAGC-3′

Assays were run over 40 cycles with the following conditions: denaturation at 95°C for 20 s, annealing at 58°C for 20 s, and elongation at 72°C for 20 s. β-actin was used to normalize the expression values of the other genes.

### Western Blot Analysis

Protein was isolated from pulverized tissue or cells from 6-well plates in ice-cold RIPA-Buffer (Beyotime, Jiangsu, China). BCA protein quantitative kit (Beyotime, Jiangsu, China) was used to evaluate the protein concentration. For Western blots, total protein were loaded in 10% SDS-PAGE and transferred onto nitrocellulose membranes. After blocking, membranes were incubated with rabbit anti-Nox2, Nox4, anti-KIM-1, anti-RIP1, anti-RIP3, anti-P-MLKL, anti-cleaved-caspase-3 antibody, anti-cleaved-caspase-8, anti-cleaved-caspase-12, and mouse anti-β-actin antibody for 18 h at 4°C, then incubated with IRDye 800-conjugated secondary antibody for 1.5 h at room temperature (1:10000, Rockland immunochemicals, Gilbertsville, PA, USA). Images were detected by Li-Cor/Odyssey infrared image system (LI-COR Biosciences, Lincoln, NE, USA) and quantified using the Image J software (NIH, Bethesda, MD, USA).

### Flow Cytometry

The extent of programmed cell death was detected by flow cytometry (BD FACSVerse, USA) using AV-FITC/PI apoptosis detection kit (Bestbio, Shanghai, China). Briefly, HK2 cells were harvested and washed twice with PBS after incubation in cell culture bottle with/without cisplatin and PA for 24 h. The cells were centrifuged at 1500 rpm/min for 5 min and stained with 5 μL Annexin V-FITC and 10 μL PI in the dark, followed by flow cytometry and quantified using FlowJo 7.6 software.

### Determination of MDA and GSH

The levels of MDA and GSH in cell or in mouse tissues were measured with a commercial kit (Jiancheng Co., Nanjing, China) according to the manufacturer’s instructions. Thiobarbituric acid reacts with MDA, degradation product of lipid peroxidation in, to generate red compound which has maximum absorbance at 532 nm. This assay is commonly called thiobarbituric acid reactive substances assay (TBARS assay). Homogenate was centrifuged at 4000 rpm/min (10 min) after incubation with TBA reagent for 40 min at 100°C. Supernatant was measured at 530 nm. 5,5-dithiobis-2-nitrobenzoic acid (DTNB) reacts with sulfhydryl compounds to generate a yellow compound, whose absorption peaks at 405 nm. GSH concentration was determined based on the absorbance of yellow compound. The homogenate was obtained and centrifuged at 3500 rpm/min for 10 min. The supernatant was reacted with DTNB and generated yellow-colored complex, which was measured at 405 nm.

### DCF Assay

DCF, the oxidation product of 2,7-dichlorodihydro-fluorescein diacetate, is a marker of cellular oxidation. Cisplatin-induced the generation of ROS as revealed by increased 2,7-dichlorodihydro-fluorescein, which was measured by fluorescence microscopy with excitation of 488 nm and emission of 525 nm after cells were incubated with DCF (10 μL/L) for 20 min at 37°C in no FBS-containing DMEM/F12 medium.

### DHE Staining

The oxidation of DHE, ethidium bound to DNA and fluoresced red, was used to estimate intracellular ROS levels. Cells were incubated with 5 μM freshly prepared DHE solution (Beyotime, Jiangsu, China) for 30 min at 37°C and then measured under fluorescence microscopy.

### Knockdown of Nox2 and Nox4 in HK2 Cells

Nox2 and Nox4 were silenced by transfection with sequence-specific or non-targeting siRNA (GenePharm, Shanghai, China)/shRNA (GeneChem Co. Ltd., Shanghai, China) using Lipofectamine^TM^ 2000 reagent (Invitrogen, Carlsbad, CA, USA) according to the manufacturer’s instructions. Briefly, for each well of a 6-well plate, 5 μl siRNA/shRNA and 5 μl lipofectamine 2000 were diluted in 200 μl Opti-DMEM separately, and incubated for 5 min at room temperature in the dark. The diluted siRNA/shRNA was combined with the diluted Lipofectamine 2000 and incubated for 20 min at room temperature. Finally, the mixture with 100 ml Opti-DMEM was applied to the cells. After incubation for 12 h, the medium was replaced with fresh DMEM supplemented with 5% FBS. Cells with Nox4 shRNA screened by puromycin were cultured in incubator at 37°C. The transfection efficiency was then evaluated using Western blotting.

### Renal Histology and Immunohistochemistry

Renal tissues were fixed in 4% PFA immediately. After dehydration, samples were embedded in paraffin. According to the manufacturer’s instruction, PAS staining was performed in Paraffin sections (3–5 μm) to assess the degree of tubulointerstitial damage and examined by light microscope (Olympus, Japan) at 200 × magnification. On PAS-stained kidney sections (*n* = 6–8), kidney damage in the cortical proximal was scored as the approximate extent of tubules that displayed tubular necrosis, cast formation, and tubular dilation as follows: 0 = normal; 1 = 10%; 2 = 10–25%; 3 = 26–50%; 4 = 51–75%; 5 = 75–95%; 6 = more than 96%. Immunohistochemistry was performed in paraffin sections using a microwave-based antigen retrieval technique (Li et al., 2012). Sections were incubated with rabbit anti-Nox4, anti-KIM-1, anti-TNF-α, and rabbit anti-F4/80 antibody overnight at 4°C. After incubation in secondary antibody and chromagen liquid DAB (3,30-diaminobenzidine tetrahydrochloride), the slides were counterstained with hematoxylin. The results were analyzed by Image Analysis System (AxioVision 4, Carl Zeiss, Jena, Germany).

### Statistical Analyses

Data are expressed as the mean ± SEM. Statistical significance was analyzed by two-tailed unpaired *t*-test or one-way analysis of variance (ANOVA), followed by Tukey *post hoc* tests using GraphPad Prism 5 software.

## Discussion

Nephrotoxicity leads to high mortality in cisplatin-treated patients with cancer, therefore identification of preventive agents for cisplatin-induced AKI is needed for clinical treatments. Here, we identified a novel TCM monomer, protocatechuic aldehyde, that protects against cisplatin-induced injury both *in vivo* and *in vitro* via inhibiting Nox-mediated oxidative stress, renal inflammation, and programmed cell death of renal tubular epithelial cells.

Protocatechuic aldehyde is a phenolic acid compound isolated from several types of Chinese herbs, including roots of miltiorrhiza, leaves of Stenolomachusanum (L.) Ching and Ilex chinensis Sims (Li et al., 2012). In several animal models, including cerebral ischemia model (Guo et al., 2016) and experimental model of sepsis (Xu et al., 2012). PA has shown pharmacological effects on inflammation and oxidative stress, which are highly correlated with pathogenesis of cisplatin nephropathy (Gao et al., 2011; Wei et al., 2013). For example, PA alleviated cerebral ischemia-reperfusion-induced oxidative injury via activating protein kinase C𝜀/Nrf2/HO-1 pathway (Guo et al., 2016). In addition, treatment of PA suppressed TNF-α-induced NF-κB phosphorylation and HMGB1 expression, attenuating inflammatory response in RAW264.7 cells (Xu et al., 2012). PA was also reported to reduce oxidative stress in sh-sy5y cells by targeting Dj-1. However, whether PA prevents cisplatin-induced AKI and the underlying mechanisms are still unknown.

In the current study, we found PA inhibited cisplatin-induced decline of renal function, renal damage, cell death and apoptosis, and inflammatory response in a dosage-dependent manner. Interestingly, accumulating evidence shows that PA possesses anti-cancer property by targeting cyclin D1-regulated cell cycle of tumor cells (Jeong and Lee, 2013; Choi et al., 2014). Here, we used three solid tumor cells lines, SMCC-7721, BEL-7402, and U87, to test the effect of PA on tumor cell viability. We found PA failed to reinforce the tumor suppressive effect of cisplatin. However, data show that PA gently promoted anti-tumor activity of cisplatin in U87 cell lines 24 h after cisplatin incubation. PA didn’t reduce anti-tumor effects of cisplatin when protected against cisplatin nephropathy, indicating PA-based therapy for cisplatin-induced nephrotoxicity in cancer patients may be effective and promising.

Our results also show that PA significantly attenuated oxidative stress by reducing ROS production in cisplatin-challenged tubular epithelial cells and kidney tissues. This may be one of the most critical mechanisms by which PA attenuates cisplatin nephropathy. To date, evidence has shown that oxidative stress plays critical roles in pathogenesis of cisplatin-induced nephrotoxicity (Humanes et al., 2012). Multiple sources for ROS in cells have been identified, including mitochondria, xanthine oxidase, cytochrome P-450, and uncoupled nitric oxide synthase (Humanes et al., 2012). NAPDH oxidases, a set of membrane-associated proteins using NADPH to transfer electrons across biological membranes and therefore generating ROS, are regarded as one of key sources for ROS in the kidney (Schreck and O’Connor, 2011). The Nox family consists of seven members (Noxs 1–5, Duox1 and 2). Nox2 and Nox4 are highly expressed within the kidney and Nox4 is known as the predominant form, which plays important roles in renal oxidative stress and kidney injury (Gill and Wilcox, 2006; Sedeek et al., 2013; Kim et al., 2016; Oh et al., 2016). Our data show that lentivirus-mediated knockdown of Nox4 *in vivo* significantly attenuated cisplatin nephropathy. In the present study, both *in vivo* and *in vitro* studies show that PA blocked cisplatin-induced ROS generation, which was evidenced by results of GSH assay, MDA assay, and ROS assay. Of note, cisplatin significantly increased protein levels of Nox4 both in cisplatin-challenged HK2 cells and cisplatin nephropathy, which was largely blocked by PA treatment. More importantly, *in vitro* data indicated that silencing Nox4, partly blocked the inhibitory effects of PA on cisplatin-upregulated levels of KIM1, cleaved-caspase-3 and production of inflammatory factors. This demonstrates that Nox4 may be the primary target in mediating the anti-oxidative stress and protective role of PA in cisplatin-induced nephrotoxicity. Additionally, it is noteworthy that Nox2 was significantly induced by cisplatin, but suppressed by PA treatment, both *in vivo* and *in vitro*; however, results generated from cisplatin-stimulated Nox2 knockdown HK2 cells showed the less important role of Nox2 in mediating the effects of PA compared with Nox4, as a critical enzyme in the injury of kidney and other organs, (Gill and Wilcox, 2006). Whether Nox2 is involved in PA treatment needs to be further validated.

We also found that PA diminished cisplatin-induced programmed cell death, especially necroptosis. As the best characterized regulated necrosis, necroptosis is known to play a critical role in cisplatin-induced AKI (Xu et al., 2015; Linkermann, 2016). Administration of Nec-1, an inhibitor for necroptosis, alleviated acute kidney injury induced by cisplatin (Tristão et al., 2012), cyclosporin A (Ouyang et al., 2012), and ischemia-reperfusion injury (Zhang et al., 2013). Emerging evidence shows that RIP1, RIP3, and downstream MLKL serve as predominant regulators in necroptosis while genetic deletion or pharmalogical inhibition of these key genes significantly reduced cisplatin-induced AKI (Linkermann et al., 2013, 2014; Xu et al., 2015). Compared with apoptosis, necroptosis plays more pivotal roles in the induction of inflammatory responses. Cell membrane collapse induces the release of damage-associated molecular patterns (DAMPs) including high-mobility group box 1 (HMGB1), heat-shock proteins, uric acid, and IL-33, which may interact with receptors (such as Toll-like receptors) and initiate downstream signaling pathways to enhance renal inflammation in a positive-feedback loop (Scaffidi et al., 2002; Vilaysane et al., 2010; He et al., 2011). However, the mechanisms by which PA blocks necroptosis is still to be determined. PA may direct target key mediators in necroptosis-regulated pathways or through indirect mechanisms in which Nox-dependent oxidative stressare invovled.

**FIGURE 11:**
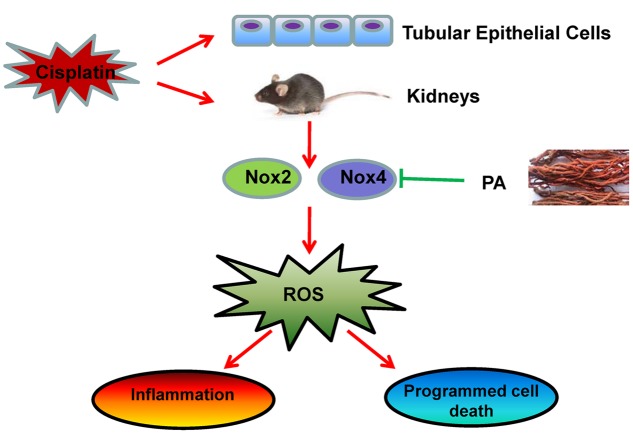
**Therapeutic effects of PA on cisplatin-induced renal injury.** PA treatment attenuates cisplatin-induced renal injury both *in vivo* and *in vitro* through Nox4-correlated mechanisms.

Collectively, as shown in **Figure 11** our study demonstrated that PA substantially alleviated the decline of renal function and renal damage while preventing renal oxidative stress, necroptosis, and consequent inflammatory response. This may be correlated with the inhibitory effect of PA on Nox4. Considering the finding that PA significantly protects against cisplatin-induced acute kidney injury without compromising the anti-cancer properties of cisplatin, it should be further explored as a preventive agent for cisplatin-treated cancer patients.

## Author Contributions

X-MM and JL designed experiments and took part in the critical revision of manuscript; LG and W-FW carried out experiments and participated in drafting of manuscript; G-LR and H-DL provided a series of experimental instructions and help; QY and X-FL conducted primary screening test of relevant drugs; TX, ZL, and B-MW analyzed experimental results; T-TM, CH, and LD analyzed and interpretation of data; YH, LZ, and XL assisted with experiments on animals.

## Conflict of Interest Statement

The authors declare that the research was conducted in the absence of any commercial or financial relationships that could be construed as a potential conflict of interest.
